# Ownership and usage of insecticide-treated bed nets after free distribution via a voucher system in two provinces of Mozambique

**DOI:** 10.1186/1475-2875-9-222

**Published:** 2010-08-04

**Authors:** Alexandre Macedo de Oliveira, Adam Wolkon, Ramesh Krishnamurthy, Marcy Erskine, Dana P Crenshaw, Jacquelin Roberts, Francisco Saúte

**Affiliations:** 1 Malaria Branch, Division of Parasitic Diseases and Malaria, Center for Global Health, U.S. Centers for Disease Control and Prevention, Atlanta, USA; 2 Center for Global Health, U.S. Centers for Disease Control and Prevention, Atlanta, USA; 3 International Federation of Red Cross and Red Crescent Societies, Geneva, Switzerland; 4 Division of Parasitic Diseases and Malaria, Center for Global Health, U.S. Centers for Disease Control and Prevention, Atlanta, USA; 5 National Malaria Control Program and Communicable Disease Division, Ministry of Health of Mozambique, Maputo, Mozambique

## Abstract

**Background:**

Insecticide-treated bed nets (ITNs) are an efficacious intervention for malaria prevention. During a national immunization campaign in Mozambique, vouchers, which were to be redeemed at a later date for free ITNs, were distributed in Manica and Sofala provinces. A survey to evaluate ITN ownership and usage post-campaign was conducted.

**Methods:**

Four districts in each province and four enumeration areas (EAs) in each district were selected using probability proportional to size. Within each EA, 32 households (HHs) were selected using a simple random sample. Interviews to assess ownership and usage were conducted in each of the selected HHs using personal digital assistants.

**Results:**

Valid interviews were completed for 947 (92.5%) (440 in Manica and 507 in Sofala) of the 1,024 selected HHs. Among participating HHs, 65.0% in Manica and 63.1% in Sofala reported that at least one child under five years of age slept in the house the previous night. HH ownership of at least one bed net of any kind was 20.6% (95% confidence interval [CI]: 7.9%-43.6%) and 35.6% (95% CI: 27.8%-44.3%) pre-campaign; and 55.1% (95% CI: 43.6%-66.1%) and 59.6 (95% CI: 42.4%-74.7%) post-campaign in Manica and Sofala, respectively. Post-campaign HH ownership of at least one ITN was 50.2% (95% CI: 41.8%-58.5%) for both provinces combined. In addition, 60.3% (95% CI: 50.6%-69.2%) of children under five years of age slept under an ITN the previous night.

**Conclusions:**

This ITN distribution increased bed net ownership and usage rates. Integration of ITN distribution with immunization campaigns presents an opportunity for reaching malaria control targets and should continue to be considered.

## Background

Malaria is endemic throughout most of Mozambique and is a major cause of morbidity and mortality. A recent study showed that 30.5% of all outpatient visits at two health facilities in southern Mozambique were due to malaria, and that most malaria cases were in young children [1]. In addition, almost 19% of all paediatric hospital deaths in this region of Mozambique were related to malaria [2]. Previous studies showed that insecticide-treated bed nets (ITNs) were effective in averting approximately one in four infant deaths in areas of intense malaria transmission once high levels of coverage among all age groups had been achieved [3,4]. In Ghana, Niger, Togo, and Zambia, free ITN distribution as part of immunization campaigns resulted in a rapid increase in ITN ownership and use [5-8].

In August and September 2005, Mozambique's Ministry of Health implemented a national immunization campaign to provide measles and polio vaccines, and vitamin A supplements to children under five years of age. Each intervention was targeted to specific age groups: 0-59 months for polio immunization, 6-59 months for vitamin A supplementation, and 9-59 months for measles immunization. This immunization campaign took place in two phases. During the first phase, August 2005, children were vaccinated against polio and measles, and received a dose of vitamin A. During the second phase, September 2005, children received a second dose of polio vaccine. In Manica and Sofala provinces (excluding the city of Beira), caregivers of children receiving the polio vaccine in September were also given a voucher that was redeemable for a free long-lasting ITN (LLIN) at a later date. According to the 2007 census, Manica and Sofala have a population of 1,412,029 (74.7% in rural areas) and 1,642,636 (61.7% in rural areas), respectively [9].

At the time of the voucher distribution, information including caregiver's name, village of residence, and child's sex and age was collected. Vouchers were adhesive and paired (two adjacent vouchers had the same number); one was put on the child's immunization card and the other on the registration book. Vouchers were limited to one per household (HH), which was defined as a woman (or guardian) and her children living together, and were to be redeemed approximately two months later for a free LLIN at fixed distribution posts in each district. Of note, pregnant women were not targeted directly by this campaign. The LLIN distribution was originally planned to be synchronized with the immunization campaign, but LLINs did not arrive in time for the immunization campaign due to limited LLIN supply in 2005.

The primary objective of the LLIN distribution campaign was to reduce morbidity and mortality due to malaria in young children. A total of 247,268 vouchers were distributed as part of this campaign. In December 2005, 358,331 LLINs were distributed to HH members upon the presentation of vouchers. Eligible HHs that did not receive a voucher in September 2005 received an LLIN during the voucher redemption phase of the campaign due to a change in Mozambican Ministry of Health policy. Originally the plan was to distribute LLINs only to mothers who presented a voucher; later the Ministry of Health determined that children should not be discriminated against if they had not been vaccinated or received a voucher. Thus, all caregivers with children under five years of age were eligible to bring their children to a distribution post to receive an LLIN. In February 2006, a cross-sectional survey was conducted to measure pre-campaign HH ownership of bed nets of any kind, post-campaign HH ownership of bed nets of any kind and ITNs, and post-campaign usage among the Roll Back Malaria (RBM) target groups (children under five years of age and pregnant women).

## Methods

### Study design and sample size

This evaluation took place from February13 through 24, 2006, approximately two months after the LLIN distribution, which took place in December 2005. The evaluation was a community-based cross-sectional survey that considered each province the domain of interest for sample size calculation purposes. The survey used a stratified three-stage cluster sample design based on the most recent census (1997) at the time, which divided the country into enumeration areas (EAs) [10]. Four districts in each of the two provinces were selected using probability proportional to size (PPS) sampling methodology. Subsequently, four EAs in each district were selected, also through PPS. Since the survey took place in the rainy season, alternate districts and EAs were also selected using PPS in case the first ones selected were inaccessible. To ensure at least 90% power to estimate the proportion of HHs that received an ITN during the campaign (estimated at 65%), with a range of plus or minus 5% and a design effect of 1.2 per province, 25 HHs per EA were randomly selected. The sample was increased by 25% to account for non-responders, meaning that 32 HHs in each EA were randomly selected.

Personal digital assistants (PDAs) (Dell Axim X50s, Dell, Austin, Texas, USA) equipped with compact flash global positioning system (GPS) units (Pharos, Torrance, California, USA) were used to conduct this survey. In the morning of each work day, all HHs in each EA were mapped. After HH mapping, a simple random sample was chosen to allow estimation of community-level HH ownership of ITNs, without regard to the presence of children under five years of age. PDAs and GPS devices were then used to navigate to the selected HHs. For these steps, GPS Sample 2.0 software, developed by the U.S. Centers for Disease Control and Prevention (CDC), was used [11].

Information on pre- and post-campaign HH ownership of bed nets of any kind and post-campaign ITN ownership and usage was collected using standardized questionnaires preprogrammed into the PDAs using Visual CE 9.1 (Syware Inc, Cambridge, Massachusetts, USA). Verbal consent from interviewees was obtained before the survey, and responses were recorded directly into the PDAs. All questions had pre-coded response categories. Replies not matching pre-coded answers were coded 'Other'.

Four teams of interviewers were trained. Teams consisted of three to five interviewers and one supervisor, all of whom were fluent in oral and written Portuguese and able to converse in other local languages. Teams mapped each EA in the morning and gathered around midday to merge HH listings and randomly select 32 HHs using the PDA. Interviews of selected HHs in an EA were conducted and completed during the afternoon of the same day.

### Definitions

For this evaluation, an ITN was defined as either an LLIN or a conventional bed net that had been treated with insecticide within the previous six months, as cyfluthrin (the insecticide most commonly used in Mozambique in 2005) is bioactive on a bed net for approximately six months. Interviewers were trained to visually inspect the bed nets and to ask questions about their treatment history, if appropriate. HH ownership of a bed net of any kind or an ITN was defined as a HH that reported having at least one of these. In addition, a bed net of any kind or an ITN was considered to have been deployed if the interviewee reported that it had been hanging the previous night or if the interviewer observed a bed net of any kind or an ITN hanging over a sleeping space. Due to the small size of some houses, persons commonly take their bed net down during the day, which is why the deployment determination relied on a combination of observation and reported use.

### Statistical analysis

The final data set was aggregated in Microsoft Access format. EPI Info 2000 (CDC, Atlanta, Georgia, USA) was used to perform initial data cleaning and Sudaan 9.0 (Research Triangle Institute, Research Triangle Park, North Carolina, USA) to calculate weighted percentages and corresponding confidence intervals (CI) incorporating the design effect of a multistage sample. Percentages presented in this manuscript are the result of weighted analysis.

The population was stratified by economic score, which was calculated according to the World Bank asset scoring for Mozambique based on the 1997 Mozambican Demographic and Health Survey [12]. Relative wealth quintiles were created by ordering all surveyed HHs by asset score and dividing them into five approximately equal groups, beginning with the lowest 20% and increasing to the highest 20%. The economic equity ratio for a given indicator was calculated as the ratio of the value of that indicator in the poorest quintile by that in the least poor quintile.

### Ethical considerations

This survey was reviewed by CDC's Institutional Review Board and deemed exempt as a nonresearch programme evaluation. Oral informed consent was obtained from each respondent.

## Results

### HH characteristics and campaign

A total of 947 HHs, 440 in Manica and 507 in Sofala, were surveyed among 1,024 selected HHs. The mean number of total persons who slept in the HH the night before the survey was 3.8 (95% CI: 3.0-4.5); the mean numbers of children under five years of age and pregnant women who slept in the HH the previous night were 0.9 (95% CI: 0.9-1.0) and 0.1 (95% CI: 0.1-0.2), respectively. In addition, 64.6% (95% CI: 61.5%-67.7%) of HHs had at least one child under five years of age sleeping in the house the previous night. In both Manica and Sofala, an alternate district had to be included because one of the initially selected districts was inaccessible. Several EAs in Sofala were inaccessible due to flooding, resulting in replacement EAs being included more often in Sofala than in Manica.

Among all 947 HHs interviewed, 48.3% (95% CI: 41.1%-55.5%) had received at least one voucher at the immunization post in September 2005. Among HHs that had received at least one voucher, 88.9% (95% CI: 81.2%-93.7%) presented at least one of those and received an LLIN. Restricting the analysis to HHs with at least one child under five years of age (the target group for this distribution), it was observed that 68.3% (95% CI: 56.5%-78.1%) of eligible HHs had received at least one voucher during the campaign. In addition, 90.4% of HHs that had at least one child under five years of age and received at least one voucher redeemed at least one of these vouchers.

Post-campaign visits by Mozambican Red Cross volunteers discussing the importance and benefits of ITN usage were conducted in 41.4% (95% CI: 25.8%-59.0%) of HHs: 26.7% (95% CI: 11.1%-51.5%) in Manica and 60.1% (95% CI: 31.7%-83.0%) in Sofala. These volunteers did not distribute any additional LLINs, but discussed malaria prevention strategies, including the use of ITNs.

### Children under five years of age

Among the 854 children under five years of age included in the survey, 407 in Manica and 447 in Sofala, the median age was 31.6 months (95% CI: 24.3-38.8) and 51.5% (95% CI: 47.8%-55.1%) were female. Of all children under five years of age included in the survey, 87.8% (95% CI: 82.8%-91.5%) went to an immunization post in September and 57.1% (95% CI: 50.5%-63.4%) received a voucher. The most common reasons for not receiving a voucher were that another child in the HH had already received a voucher or that no vouchers were available at the immunization post at the time of their visit. Finally, 48.9% (95% CI: 40.3%-57.7%) of caregivers of children under five years of age went to distribution posts and received an LLIN during the distribution phase.

### Bed net characteristics

In Manica, information on 309 bed nets of any kind was collected. Of these, 279 (90.4%; 95% CI: 82.2%-95.0%) were ITNs. Among ITNs, 256 (91.2%; 95% CI: 80.5%-96.3%) were campaign LLINs, and 23 (8.8%; 95% CI: 3.7%-19.6%) were ITNs of other origin. Of all ITNs in Manica, 91.0% (95% CI: 85.3%-94.6%) were reported to have been hanging the night before the survey. In Sofala, 369 bed nets of any kind were identified. Of those bed nets, 279 (75.9%; 95% CI: 70.0%-80.8%) were ITNs. Among ITNs, 249 (89.0%; 95% CI: 84.0%-92.5%) were campaign LLINs, and 30 (11.0%; 95% CI: 7.5%-16.0%) were ITNs of other origin. In Sofala, 96.6% (95% CI: 92.6%-98.5%) of ITNs were reported to have been hanging the night before the survey.

### ITN ownership, usage, and equity

#### Manica Province

**Figure 1:**
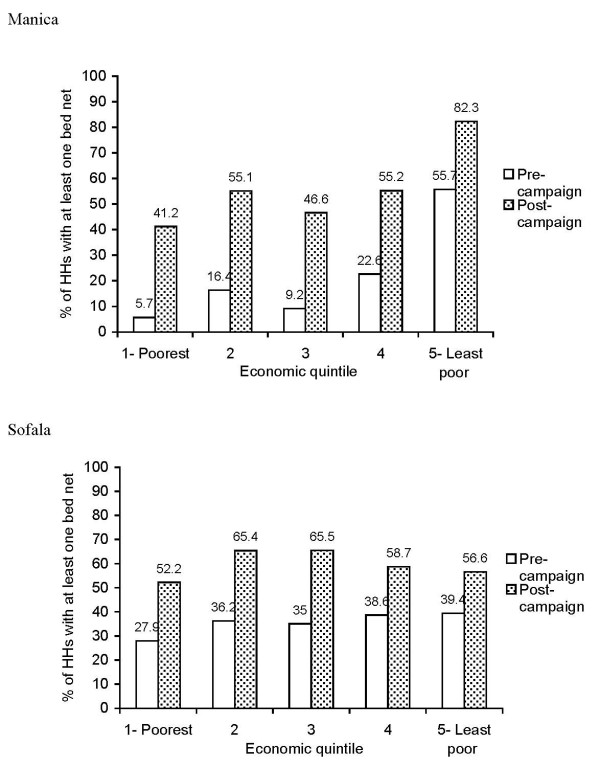
**Pre- and post-campaign household (HH) ownership of at least one bed net of any kind by economic quintile in Manica and Sofala provinces, Mozambique, 2006**.

Pre-campaign HH ownership of at least one bed net of any kind was 20.6% (95% CI: 8.0%-43.6%) compared with post-campaign ownership of 55.1% (95% CI: 43.6%-66.1%) (p = 0.0012). Pre-campaign HH ownership of at least one bed net of any kind was lower in the poorest economic quintile (5.7%; 95% CI: 1.3%-21.8%) compared with the least poor quintile (55.7%; 95% CI: 43.0%-67.8%): an equity ratio of 0.10. Additionally, post-campaign HH ownership of at least one bed net of any kind in the poorest quintile was 41.2% (95% CI: 19.3%-67.4%) compared with 82.3% (95% CI: 68.9%-90.7%) in the least poor quintile, resulting in an equity ratio of 0.50 (Figure 1).

Post-campaign HH ownership of an ITN was 51.5% (95% CI: 41.3-61.5), varying from 40.3% in the poorest quintile to 74.3% in the least poor quintile. The equity ratio for post-campaign ITN ownership was 0.54 (see Table 1). In addition, 48.1% (95% CI: 36.9%-59.5%) of HHs reported at least one ITN hanging the previous night. When only HHs that owned at least one ITN were considered, this estimate was 89.2% (95% CI: 84.0%-92.9%).

**Table 1:** Post-campaign HH ownership (%) of at least one ITN by economic quintile in Manica and Sofala provinces, Mozambique, 2006.

Economic quintile	HH ownership (%) (95% confidence interval [CI])	Number of HHs (n)
Manica		
All HHs	51.5 (41.3-61.5)	440
Quintile 1 (poorest quintile) HHs	40.3 (19.9-64.7)	96
Quintile 2 HHs	54.2 (39.7-68.1)	107
Quintile 3 HHs	40.9 (25.7-58.1)	83
Quintile 4 HHs	51.2 (42.6-59.7)	89
Quintile 5 (least poor quintile) HHs	74.3 (62.0-83.8)	65
Equity ratio (ratio)	0.54	
Sofala		
All HHs	48.5 (34.8-62.4)	507
Quintile 1 (poorest quintile) HHs	42.9 (24.8-63.1)	95
Quintile 2 HHs	48.0 (31.3-65.3)	82
Quintile 3 HHs	55.8 (37.1-72.9)	106
Quintile 4 HHs	50.0 (24.5-75.5)	100
Quintile 5 (least poor quintile) HHs	45.3 (38.3-52.5)	124
Equity ratio (ratio)	0.95	
Total	50.2 (41.8-58.5)	

Usage of ITNs by children under five years of age following the distribution was 59.3% (95% CI: 54.4%-63.9%): 48.4% (95% CI: 26.9%-70.5%) in the poorest quintile and 71.9% (95% CI: 65.6%-77.5%) in the least poor quintile. The equity ratio of usage by children under five years of age ITN was 0.67. When only children under five years of age who lived in a HH that owned at least one ITN were considered, 96.7% (95% CI: 86.0%-99.3%) slept under an ITN the night prior to the survey.

#### Sofala Province

Pre-campaign HH ownership of at least one bed net of any kind was 35.6% (95% CI: 27.8%-44.3%) compared with post-campaign ownership of 59.6% (95% CI: 42.4%-74.7%) (p = 0.0008). Pre-campaign HH ownership of at least one bed net of any kind was 27.9% (95% CI: 20.3%-37.0%) in the poorest economic quintile and 39.4% (95% CI: 27.8%-52.2%) in the least poor quintile, an equity ratio of 0.71. Post-campaign HH ownership of at least one bed net of any kind varied from 52.2% (95% CI: 22.7%-80.3%) in the poorest quintile to 56.6% (95% CI: 45.2%-67.2%) in the least poor quintile. The resulting post-campaign equity ratio for this indicator increased to 0.92 (Figure 1).

Post-campaign HH ownership of at least one ITN was 48.5% (34.8%-62.4%): 42.9% in the poorest quintile and 45.3% in the least poor quintile. The resulting equity ratio was 0.95. These results are summarized in Table 1. During the evaluation, 47.6% (95% CI: 33.8%-61.7%) of HHs reported at least one ITN hanging the previous night; this estimate was 92.4% (95% CI: 85.9%-96.0%) when only HHs that owned at least one ITN were considered.

Post-campaign ITN usage by children under five years of age was 61.6% (95% CI: 41.2%-78.6%): 54.5% (95% CI: 20.7%-84.6%) in the poorest quintile and 61.6% (95% CI: 54.3%-68.3%) in the least poor quintile, an equity ratio of 0.88. When only children under five years of age who lived in a HH that owned at least one ITN were considered, 95.7% (95% CI: 93.7%-97.1%) slept under an ITN the night prior to the survey.

#### Combined data for both provinces

When data from both provinces are combined, HH ownership of at least one bed net of any kind increased from 27.2% (95% CI: 17.9%-37.0%) pre-campaign to 57.1% (95% CI: 47.1%-66.5%) post-campaign, a difference that was statically significant (p = 0.0002). Post-campaign HH ownership of at least one ITN, on the other hand, was 50.2% (95% CI: 41.8%-58.5%) and ITN usage by children under five years of age was 60.3% (95% CI: 50.6%-69.2%) for both provinces combined (Table 2).

**Table 2:** ITN usage (%) by children under five years of age per district in Manica and Soala provinces, Mozambique, 2006.

District	ITN usage (%) (95% CI)	Number of children under five years of age (n)
Manica		
Gondola	64.5 (54.4-73.5)	115
Manica	57.5 (22.3-86.5)	93
Mossurize	59.8 (41.4-75.9)	96
Sussudenga	55.6 (40.3-70.0)	103
Subtotal Manica	59.3 (44.7-72.4)	407
Sofala		
Caia	46.9 (32.3-62.0)	110
Chemba	82.3 (75.0-87.7)	108
Dondo	50.5 (44.8-56.3)	103
Marromeu	64.4 (35.3-85.7)	126
Subtotal Sofala	61.6 (50.1-72.0)	447
Total	60.3 (50.6-69.2)	854

### Pregnant women

Information on a total of 100 pregnant women was included in our survey, 46 from Manica and 54 from Sofala. In Manica, it was reported that 33.9% (95% CI: 25.8%-43.0%) of pregnant women slept under an ITN the previous night, while in Sofala this percentage was 43.1% (95% CI: 33.8%-53.0%). For both provinces combined, this estimate was 38.0% (95% CI: 30.4%-46.1%).

## Discussion

This survey was designed to evaluate ITN ownership, usage, and equity following the distribution of free LLINs in Manica and Sofala provinces, central Mozambique. Vouchers redeemable for free LLINs were distributed as part of the second phase of an immunization campaign and were to be redeemed for free LLINs approximately two months afterwards. By providing free LLINs to children under five years of age, the campaign was intended to increase HH ownership and usage of ITNs in these two provinces. These results show increased HH ownership and usage by children under five years of age, 50.2% and 60.3%, respectively, when data for both provinces are combined. The campaign also served to improve equity of ITN ownership in this region of Mozambique.

This survey found that 87.8% of children under five years of age went to an immunization post in September 2005, but only 57% of children received vouchers for LLINs because, among other reasons, the campaign limited the number of vouchers to one per HH. Approximately 50% of children under five years of age received an LLIN. Using vouchers may achieve lower coverage rates compared with direct ITN distribution during immunization campaigns, because the extra step of voucher exchange at an ITN redemption post is required [8,13]. In addition, limited financial resources often do not allow for voucher redemption sites to be as numerous as immunization posts during the immunization campaign, meaning that caregivers needed to go greater distances to reach a redemption post. Despite these limitations, free distribution of ITNs using vouchers is a commonly used alternative in instances when bed nets are not available at the time of the immunization campaign or it is not feasible to coordinate simultaneously the logistics of both immunization campaigns and bed net distribution [5].

HH ownership of bed nets of any kind increased from 20.6% to 55.1% in Manica, with an improved equity ratio. In Sofala, HH ownership of any bed nets increased to 59.6%, also with a higher equity ratio. These proportions are lower than those observed in Niger (>80%), where similar surveys were conducted one and nine months after an immunization campaign and bed net distribution [8]. The difference between the results of these campaigns can in part be explained by the higher proportion of HHs with a child under five years of age in Niger (75%) compared with Mozambique (65%). In addition, Niger was reported to have a pre-existing bed net usage culture, which might have contributed to increased awareness and interest in having an LLIN [8]. Nonetheless, this difference highlights one of the inherent limitations of ITN distribution via mass immunization campaigns, which is the targeting of a subset of HHs, i.e., those with children eligible for the vaccination [5]. There is no doubt that higher levels of ITN ownership and usage can be achieved with universal distributions. As national malaria control programmes move towards universal access to ITNs, innovative strategies will need to be put in place to attain and maintain high levels of coverage among different age groups [14,15].

This campaign in Mozambique was successful in increasing HH ownership of ITNs to 50.2% in both provinces combined. It was not possible to determine the insecticide treatment status of bed nets present in the HH prior to the campaign. However, considering the proportions of ITNs among non-campaign bed nets at the time of the survey (53 ITNs among 173 non-campaign bed nets or 30.6%), the pre-campaign HH ownership of ITNs can be estimated at 8.3% (27.2% times 30.6%), lower than what was observed post-campaign. These findings are consistent with previous studies in the literature, which suggest that integrated campaigns to distribute ITNs are able to rapidly increase HH ownership of ITNs [5,7,13]. However, in order to maintain and further increase these high ownership rates, it is recommended that other strategies, such as ITN distribution at antenatal clinics and during children's regular immunization visits, be undertaken as complementary efforts [16,17].

The results of this survey show that HH ownership was achieved more equitably in Sofala than in Manica. This difference in equity could be related to the fact that pre-campaign HH ownership of bed nets of any kind was more equitable in Sofala (35.6%, equity ratio = 0.71) than in Manica (20.6%, equity ratio = 0.10). In addition, this finding could also have resulted from differences in the distribution between the two provinces, such as the higher rate of post-distribution visits by Mozambican Red Cross volunteers in Sofala to promote usage, which might have contributed to higher rates of LLIN retention.

When ITN usage by children under five years of age (campaign and RBM target group) was considered, the usage rate for both provinces combined was above the 60% initial Abuja target for vulnerable populations. In addition, the adherence rate (children sleeping under an ITN given at least one ITN in the HH) was greater than 95%. Different factors may have contributed to this finding. First, one can expect that people who made a trip to the voucher redemption post were more aware of the benefits of sleeping under an ITN and consequently more prone to adhere to such behaviour. Second, this survey was conducted late in the rainy season, with a likely increase in mosquito population, a factor known to increase usage of bed nets [18,19]. A survey in Togo during the rainy season, nine months after LLIN distribution, found an increased usage rate, 69.5% versus 52.8% in the dry season, in HHs with at least one ITN (Wolkon A, unpublished data). Third, this higher adherence rate could in part be attributed to the work done by volunteers involved in social mobilization, voucher and LLIN distributions, and post-campaign follow-up visits.

This survey has several limitations. First, given the cross-sectional design of the survey, it was not possible to understand how ITN ownership and usage may vary over time. Second, our evaluation was focused on the end results of the campaign, i.e., ITN ownership and usage, and provided little information about improving the overall process of the campaign. Analysis of the campaign itself might have yielded important lessons for improving future distribution strategies. Heavy rains made some of the initially selected EAs inaccessible, especially in Sofala. This might have contributed to an overestimation of our indicators, since reachable areas, which could coincide with areas that also had an easier access during the distribution efforts, were more easily accessible. Finally, because children under five years of age were eligible to collect an LLIN at the distribution post even without presenting a voucher, it was not possible to assess the effect a voucher-based bed net distribution might have had on reducing LLIN uptake by the target population.

In conclusion, this voucher-based LLIN distribution in Sofala and Manica appears to have been an effective strategy to rapidly scale up ITN ownership and usage. The figures for HH ownership of ITNs, although encouraging, are below the then-Abuja targets, and efforts to achieve higher rates should be considered in the future. The overall project did achieve the then-Abuja target for ITN usage by children under five years of age when the provinces were combined, but achieved this target only for Sofala when data from each province are analyzed separately. Integration of ITN distribution with immunization campaigns presents an important opportunity for reaching malaria control strategies among young children. Whenever feasible, bed nets should be distributed directly at the time of vaccine administration to help maximize immediate ownership and usage indicators among children under five years of age. As countries move toward universal access to ITNs, other strategies may need to be developed to reach older population groups [16,20].

## Competing interests

The authors declare that they have no competing interests.

## Authors' contributions

AMO and AW participated in the design of the survey, data collection in the field, analysis of the data, and writing of the manuscript. RK assisted in data collection and management. ME participated in the design of the survey and supervised field work. DPC programmed personal digital assistants. JR performed statistical analysis. FS participated in the design of the survey. All authors revised and approved the final manuscript.
